# Federated learning framework integrating REFINED CNN and Deep Regression Forests

**DOI:** 10.1093/bioadv/vbad036

**Published:** 2023-03-22

**Authors:** Daniel Nolte, Omid Bazgir, Souparno Ghosh, Ranadip Pal

**Affiliations:** Department of Electrical and Computer Engineering, Texas Tech University, Lubbock, TX 79409, USA; Genentech, South San Francisco, CA 94080, USA; Department of Statistics, University of Nebraska—Lincoln, Lincoln, NB 68588, USA; Department of Electrical and Computer Engineering, Texas Tech University, Lubbock, TX 79409, USA

## Abstract

**Summary:**

Predictive learning from medical data incurs additional challenge due to concerns over privacy and security of personal data. Federated learning, intentionally structured to preserve high level of privacy, is emerging to be an attractive way to generate cross-silo predictions in medical scenarios. However, the impact of severe population-level heterogeneity on federated learners is not well explored. In this article, we propose a methodology to detect presence of population heterogeneity in federated settings and propose a solution to handle such heterogeneity by developing a federated version of Deep Regression Forests. Additionally, we demonstrate that the recently conceptualized REpresentation of Features as Images with NEighborhood Dependencies CNN framework can be combined with the proposed Federated Deep Regression Forests to provide improved performance as compared to existing approaches.

**Availability and implementation:**

The Python source code for reproducing the main results are available on GitHub: https://github.com/DanielNolte/FederatedDeepRegressionForests.

**Contact:**

ranadip.pal@ttu.edu

**Supplementary information:**

Supplementary data are available at *Bioinformatics Advances* online.

## 1 Introduction

With the recent imposition of regulations to safeguard consumer data (e.g. General Data Protection Regulation), interest in developing privacy preserving predictive models has increased substantially. Storage, distribution and utilization of medical data require compliance with regulations (e.g. Health Insurance Portability and Accountability Act), which often prohibit training effective predictive models on centralized data that pools raw medical data distributed among multiple hospitals. Recently developed federated learning (FL) technique allows efficient learning from such distributed data without centralizing it. Broadly speaking, FL only requires exchange of model updates among various computing entities allowing for the data to stay with the data owners and thereby preserving the basic privacy requirement associated with data transmission. In addition, FL allows the shareable model updates to be secured as well, thereby providing additional privacy, while also fitting a predictive model to the distributed data. Due to such in-built privacy protecting features, FL is being already adopted on real consumer data (Dayan *et al.*, 2021; Pati *et al.*, 2021, 2022a; Yang *et al.*, 2018). Dayan *et al.* (2021), e.g. trained a federated deep and cross network with a real federation consisting of 20 institutions from around the world for predicting future oxygen requirements of symptomatic COVID-19 patients. They showed that the federated model improved the AUC 16% on average compared to the client alternative models that were trained solely on their local data. Deployment of FL is also gaining traction in medical scenarios (Rieke *et al.*, 2020; Sheller *et al.*, 2019, 2020; Xu *et al.*, 2021) and multiple open-source tools have been released to assist researchers (Katti *et al.*, 2022; Pati *et al.*, 2022b; Reina *et al.*, 2021). For instance, Sheller *et al.* (2020) trained a federated U-net for brain tumor detection and showed FL’s ability on privacy-sensitive applications to perform nearly as well as the case where all the data were shared to a centralized location for traditional model training. On the other hand, Roth *et al.* (2020) created a real-world federation of seven clinical institutions to train a federated CNN for mammography breast density classification. Despite large differences among the institutions’ local datasets, they showed that the federated model, on average, performed considerably better as compared to the client-specific counterparts. For a systematic review of FL in the medical context, we direct the audience to Pfitzner *et al.* (2021).

In this article, we consider the generation of a FL framework with applications in medical environments where the clients can be different hospitals or research laboratories. We assume that although the clients are not willing to share raw medical data but are keen on building an effective predictive model that learns from data managed by each client. For instance, developing a federated drug efficacy prediction model from genomic data where each client (research laboratories, pharmaceutical companies etc.) has tested the efficacy of a common set of drugs on their in-house, potentially non-overlapping, cell-line samples could generate more precise prediction as compared to separate predictive models developed by individual clients. Thus, for a successful FL framework, we need to demonstrate (i) the predictive performance of the federated learner is on an average better than individual client models and initial server model; (ii) the chosen predictive model that facilitates FL is itself better (in predictive sense) than other existing predictive models that are amenable to federated setting; (iii) privacy of the client data is maintained, in the sense that, there is no leakage of raw data among clients and between clients and server; and (iv) robust performance of the federated learner under various scenarios of disjointedness among client datasets—for instance, imagine that each client has tested the efficacy of drug compounds on unique cell-lines that each client has access to thereby yielding a set of non-overlapping observational units across the client base. However, since cell-line information cannot be shared among clients, fitting a global model is infeasible.

Since typical aggregation methods rely on an iterative learning scheme (McMahan *et al.*, 2017; Li *et al.*, 2020; Wang *et al.*, 2020), such as SGD, where after each iteration the models can be aggregated using gradient updates, not all models can be deployed in the federated setting. Convolutional neural networks and similar gradient-based deep networks, on the other hand, are more amenable to FL frameworks (Dayan *et al.*, 2021; Feki *et al.*, 2021; Roth *et al.*, 2020). However, biological datasets often come with non-image inputs, such as transcriptomic features, which precludes deployment of vanilla CNN because a naive lexicographic 2D mapping of transcriptomic features will fail to maintain neighborhood structures that are important for CNN type models to perform. Thus, in this article, we demonstrate how to integrate recently proposed REpresentation of Features as Images using NEighborhood Dependencies (REFINED) CNN framework (Bazgir *et al.*, 2020) that arranges high-dimensional vectors in a compact image form thereby making them conducive for CNN-based deep learning in the federated setting. To further bolster the regression performance, we arm the prediction head of foregoing REFINED-CNN with Deep Regression Forest (DRF) (Shen *et al.*, 2018). DRFs connect a neural network model with a forest of probabilistic trees by using the preceding model’s outputs as the routing probabilities of the decision nodes for each of the samples thus combining the benefits of neural network feature extraction with the robustness of prediction obtained from the ensemble of trees.

More specifically, inspired by federated deep neural decision forest of Sjöberg *et al.* (2019), we demonstrate the use of REFINED with federated DRFs on non-image medical data for improved accuracy in the cross-silo horizontal FL scenarios. The proposed method first maps high-dimensional feature vectors into images then uses these images to train a federated DRF. In doing so, we make methodological contributions in demonstrating that (i) CNN can be brought to bear in federated setting even when the federation is dealing with non-image data and (ii) Sjöberg *et al.* (2019) can be extended to federated DRFs as well. Our framework’s effectiveness on improving prediction accuracy is demonstrated on two biological datasets. Results show that the REFINED federated DRF improves federated predictive performance on both datasets under different choices of the size of federation. We also illustrate that federated models outperform the separately trained local client models by a considerable margin. We further illustrate the robust performance of REFINED federated DRF in the presence of heterogeneous client datasets.

## 2 Methods

### 2.1 Federated learning

FL is a relatively new learning paradigm introduced in 2016 (McMahan *et al.*, 2017). FL has gained considerable attention since it allows distributed learning without sharing raw, potentially confidential, data thereby offering an additional layer of protection toward data privacy.

**Fig. 1.**
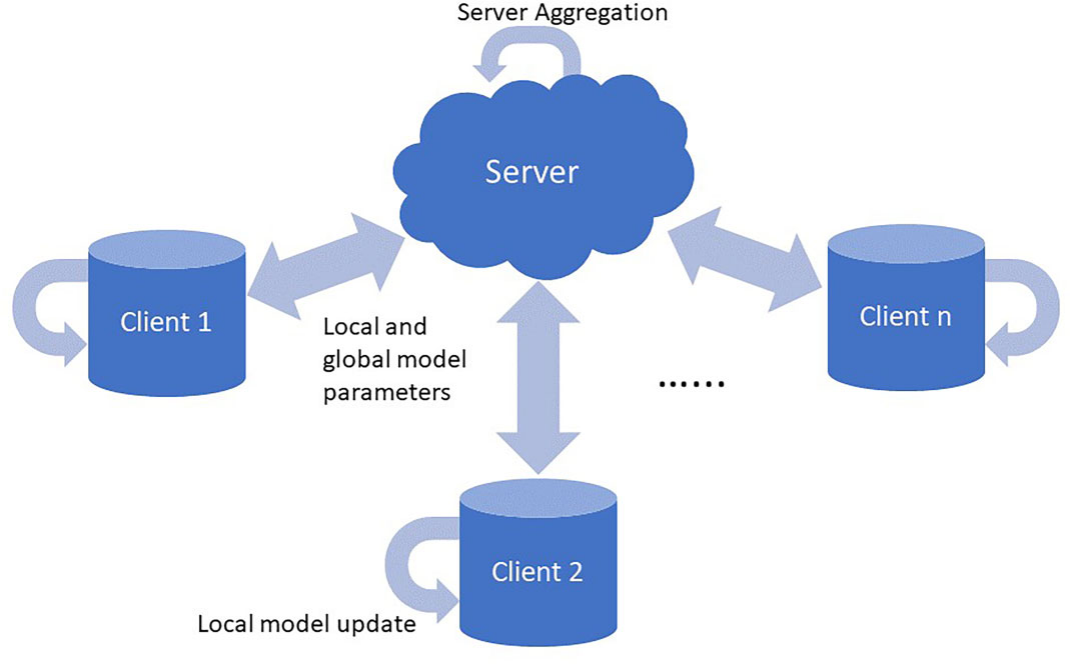
FL process with server aggregation, local and global model exchanges and local client model updates

Figure 1 depicts the typical FL process where a federation of a server and *n* clients is formed with each client trying to protect the confidentiality of its own raw data, but is willing to participate in a distributed learning process with the expectation that an aggregated model—developed by sharing non-confidential information across the federation—will have better performance than individual client models. In each training round, clients are selected for a model update where they each initialize their model from the global model received from the server, then train their model on their private data. Once each selected client finishes training, they send their updated model parameters or model gradients back to the server who aggregates the updates from the selected clients into the new global model using Federated Averaging (McMahan *et al.*, 2017). This process repeats itself until suitable model performance is achieved or a valid stopping criterion is reached. To jump start the training process, it can be assumed that the server has some data to estimate the initial model, either through publicly available datasets or through client data that is not privacy sensitive (Ding *et al.*, 2022; Li and Wang, 2019; Tzinis *et al.*, 2021). Such pre-training of an initial model on proxy data has been shown to increase the predictive accuracy of the federated learner and reduce the number of aggregations required to reach a target error (Chen *et al.*, 2022; Nguyen *et al.*, 2022; Zhao *et al.*, 2018). In FL, the data can be partitioned in multiple ways, the most popular being horizontal FL and vertical FL. In horizontal FL, the clients share the same features space but have different samples. Whereas with vertical FL the clients have the same sample space, but differing features for each client. In this article, we are exclusively working in the horizontal setting, where clients have different samples sharing the same features.

### 2.2 REFINED

Conventional CNN is an effective tool when the predictors come in the form of images because, architecturally, CNN exploits the spatial correlation among neighbors in images during the convolution operation which reduce the number of free model parameters as compared to a fully connected network. However, both 1D and 2D CNNs are not conducive for datasets consisting of high-dimensional vectors that do not display any natural ordering. Medical datasets often have such non-image features (e.g. transcriptomics information) where a naive lexicographic 2D mapping will fail to maintain spatial correlations among features thereby precluding deployment of vanilla CNN type models. Recently we developed the REpresentation of Features as Images with NEighborhood Dependencies (REFINED) CNN framework that arranges high-dimensional vectors into a compact image form that can be subsequently processed via CNN-based deep learning protocols (Bazgir *et al.*, 2020). REFINED starts by obtaining the pairwise distance between features from the data and then applies multi-dimensional scaling (MDS) to initialize a feature map that is fed into Bayesian MDS, which iteratively estimates the feature locations with a constraint that no two features can share the same location. REFINED CNN and its robustified version were shown to perform better than competing approaches in multiple biological prediction scenarios (Bazgir *et al.*, 2020, 2021).

In this article, we use the foregoing REFINED technique to map high-dimensional vectors, encoding biological and chemical information, into images before model training. An *a-priori* REFINED map is generated based on the dataset available with the server and shared with all clients before the federated training kicks in.

### 2.3 Federated DRFs

**Fig. 2.**
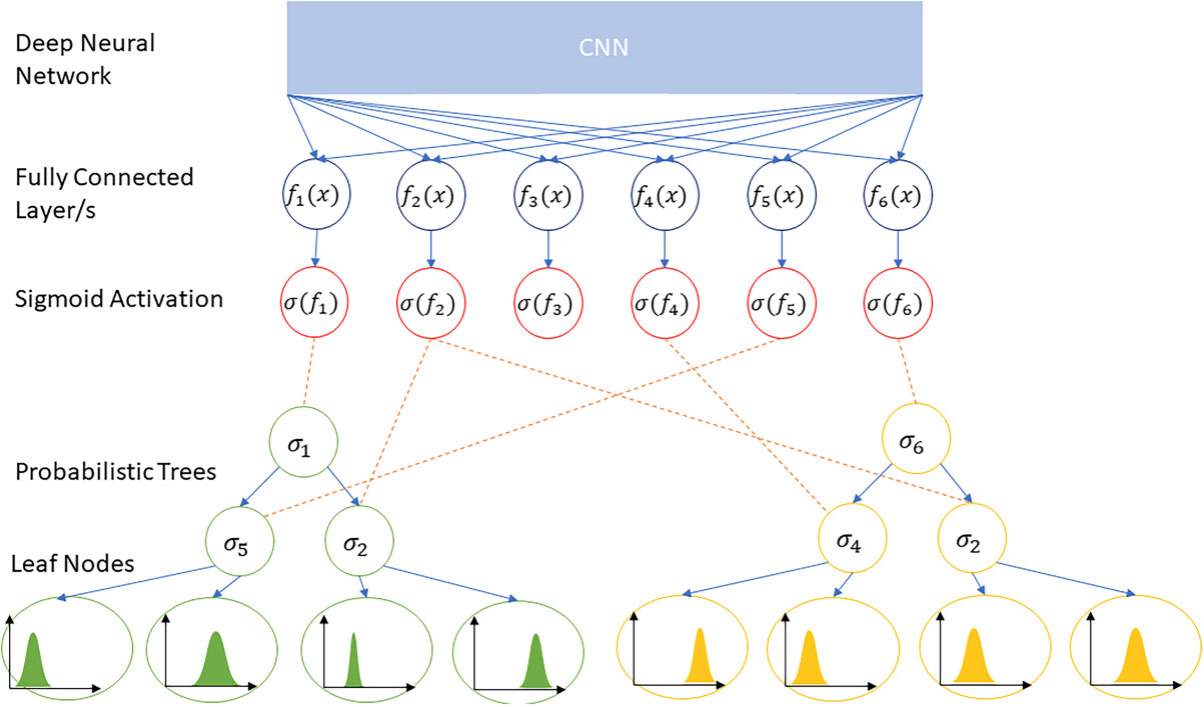
Example DRF with six feature outputs and two trees each with a depth of 3

DRFs connect deep neural networks to a forest of trees with probabilistic routing of samples. The deep networks, typically CNNs, are used to extract rich representational features, which are then transformed into probabilities through a sigmoid function, and used for the routing of a sample through the tree. Each leaf node has a probability density function, typically specified via Gaussian distribution, over the target space. The predictions of the trees are the sum of the expected values of the leaf node distributions of the trees weighted by the probabilities of the sample reaching that leaf. Figure 2 shows an example DRF with a CNN network, six feature outputs and two trees each with a depth of 3. For an ensemble of trees, the forest predictions are simply the average of the predictions from each tree.

For training a DRF, both the model parameters and leaf node distribution parameters are learned from the data. The model parameters are randomly initialized and the leaf node distributions are initialized with a Normal(1, *I*) distribution, where *I* is an identity matrix of appropriate dimension. Then, holding the leaf node distribution parameters fixed, the network parameters are updated through typical backpropagation. After the network parameters are updated, they are held constant and the leaf node distribution parameters are iteratively updated through variational bounding. Shen *et al.* showed that the soft split loss function associated with tree nodes are differentiable with respect to the network model parameters and the gradient of the loss can be used in typical backpropagation to update the model parameters (Kontschieder *et al.*, 2015). This alternating optimization scheme allows DRFs to learn both the model parameters and leaf node distributions via a coherent training pipeline.

In this article, we make DRFs amenable to federated setting by aggregating all the model parameters and leaf node parameters using typical federated averaging aggregation (McMahan *et al.*, 2017). More precisely, we allow each client to perform one epoch of network parameter updates using their local data, followed by the leaf node parameter update, again on their local data, before sending the updates back to the server. The server then aggregates the model parameters by taking appropriate data weighted average of each of the model parameters from each of the clients. These updated parameters are then sent back to the clients.

## 3 Results

Let $D={\left\{D_{i}\right\}}_{i=1}^{N}$ denote the available information on *N* samples. The dataset was partitioned into training set ($D_{T}$), validation set ($D_{V}$) and test set ($D_{H}$). One server and *n* clients were available to deploy the federated framework. Only the server had access to $D_{V}$ and an initial training dataset $D_{T}^{\left(I\right)}$ to the initialize federated learner. The clients had access to portions of $D_{T}^{\left(-I\right)}=D_{T}-D_{T}^{\left(-I\right)}$ for their own training purposes. First, the data was randomly split between the clients, then a robustness analysis was performed to show the performance of our method in different realistic scenarios where drug–target metadata were brought into play to split the data among clients in a more cognizant fashion. $D_{H}$ was held outside the federated loop to test the predictive performances of the competing models.

Normalized root mean square error (NRMSE) and Pearson correlation coefficient (PCC) were used to compare predictive performances of each of the models considered herein. The NRMSE is defined as: where *y* is the target data, $\hat{y}$ is the predicted values and $\bar{y}$ is the sample mean of the target variable. The NRMSE is used as it allows comparison of all models with the baseline average value prediction model.


(1) $$\mathrm{NRMSE}=\sqrt{\frac{1/|j\in D_{H}|\sum_{j\in D_{H}}{\left(y_{j}-{\hat{y}}_{j}\right)}^{2}}{1/|j\in D_{H}|\sum_{j\in D_{H}}{\left(y_{j}-{\bar{y}}_{j}\right)}^{2}}},$$

The PCC is a measure of collinearity between the predicted and true target values and is often used to measure the goodness of fit of a model up to a linear bias. However, since PCC is scale and offset invariant, we use NRMSE to provide further insight into the models goodness of fit. Consequently, higher PCC coupled with low NRMSE will imply that predicted values are not only collinear with the target values, but also the former are, on average, tightly spread around their targets. Evidently, models with uniformly high PCC and low NRMSE will be preferred. Since our considered scenario deals with medical data distributed among research labs or hospitals, i.e. cross-silo, we assume the federation will contain a server and a small set of clients who have reliable connection and the required computational resources to train local models independently. Additionally, we will assume that the entire set of clients participate in each training round thereby alleviating an additional layer of complexity induced when millions of low-powered devices (e.g. cell phones) partake in the federation that requires a randomly selected subset of clients to participate in each training round. To ascertain the impact of federation size on prediction performance, for each model, hyper parameters, and optimizers, we varied the number of clients in the federation by assigning n∈{3,5,10,15,20}. Further information on the simulation setup, datasets, preprocessing and candidate models can be found in the Supplementary Material.

### 3.1 CCLE

**Fig. 3.**
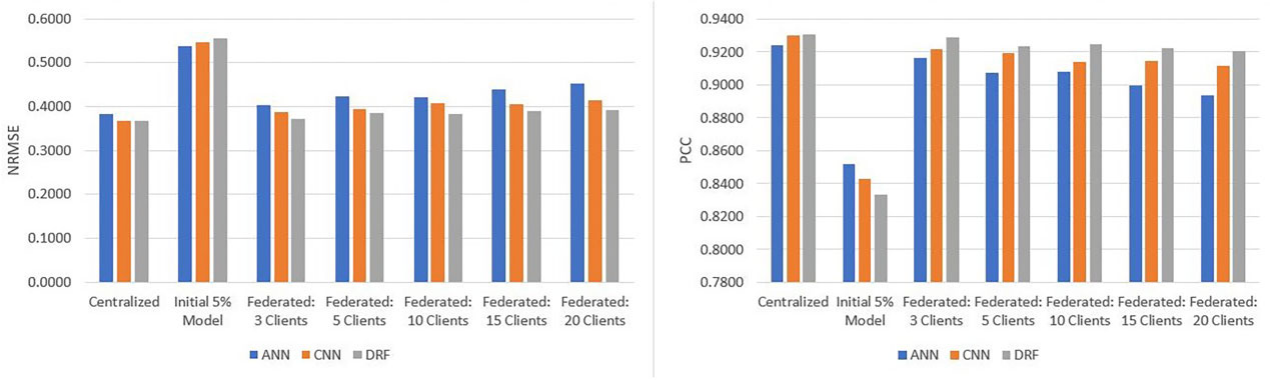
Average CCLE test results over three different data partitions with ANN, REFINED-CNN and REFINED-DRF on CCLE in terms of NRMSE and PCC

The cancer cell line encyclopedia (CCLE) dataset consists of responses elicited by 24 anticancer drugs screened on 504 different cancer cell-lines with known genomic information. Further details on the datasets are provided in the Supplementary Material. Results pertaining to centralized, initial and federated setting are all reported as the average performance across the three different random data splits. The NRMSEs and PCCs for each model for the CCLE dataset are shown in Figure 3 (more detailed results, in tabular format, are offered in Supplementary Table S2). Figure 3 demonstrates that REFINED-DRF consistently achieved better performance in CCLE under all federation sizes and in the centralized setting as well. The only case where the REFINED-DRF was outperformed was on the initial 5% model where the ANN had a marginally better performance. REFINED-CNN also outperformed the ANN in all centralized and federated cases except the 5% initial model. However, given the predictive discrepancy between initial model and the centralized benchmark, the former cannot be claimed as an ‘adequate’ model in the first place.

On all federated models, as the number of clients increased, the predictive performance decreased, with the three-client case being almost as good as the centralized benchmark. However, interestingly, the performance gap between REFINED-DRF and ANN increased noticeably as the number of clients increased. As shown in Figure 3, the ANN with 3(20) clients achieved an NRMSE of 0.40 (0.45) and a PCC of 0.916 (0.894), whereas the REFINED-DRF with 3(20) clients achieved an NRMSE of 0.37 (0.39) and a PCC of 0.929 (0.921) PCC. Thus, for three-client case, REFINED-DRF improved NRMSE by 8% as compared to ANN. But, for 20-client case, REFINED-DRF improved NRMSE by 15% as compared to ANN. Similar calculation for PCC revealed REFINED-DRF improved PCC by 1.5% (3%) in 3 (20)-client case as compared to ANN. Evidently, as the number of clients increased, the performance of ANN appeared to deteriorate at a faster rate as compared to the predictive degradation of REFINED-DRF.

To assess the utility of federation, we considered the baseline models where all three competing federated models were independently trained and tested on each of the local client datasets separately. All client sizes were trained and tested on the same sets as the federated simulations. The client models used the same initial 5% REFINED mapping as the federated simulations for fair comparison. The models also used the same early stopping and learning rate scheduling, but on a 10% validation subset of their data. Additionally, since the training datasets are much smaller, the model complexities were reduced by removing layers and decreasing the batch size to 12, allowing for better individual client model performance. For the CNN and DRF, the last CNN layers for both of the drug and cell arms were removed. Furthermore, each CNN and DRF model had only one hidden dense layer. For the ANN, the first two hidden layers were removed. The average test results across the three data partitions for each client size are shown in Supplementary Table S3. We observe that the individual client models perform significantly worse than their federated counterparts. ANN outperformed its competitors under all client size scenarios considered herein. However, on average, the federated ANN showed ∼13% (4%) improvement in NRMSE (PCC) as compared to the baseline ANN. Federated REFINED-DRF, on the other hand, showed ∼24% (7%) improvement in NRMSE (PCC) as compared to its baseline counterpart and was able to greatly decrease the generalization error of the models utilizing FL. The average generalization error improvement for the ANN, CNN and DRF NRMSEs, respectively, are 13.6%, 20.6% and 24.4% for the federated models compared to the individual client models.

### 3.2 NCI-60

**Fig. 4.**
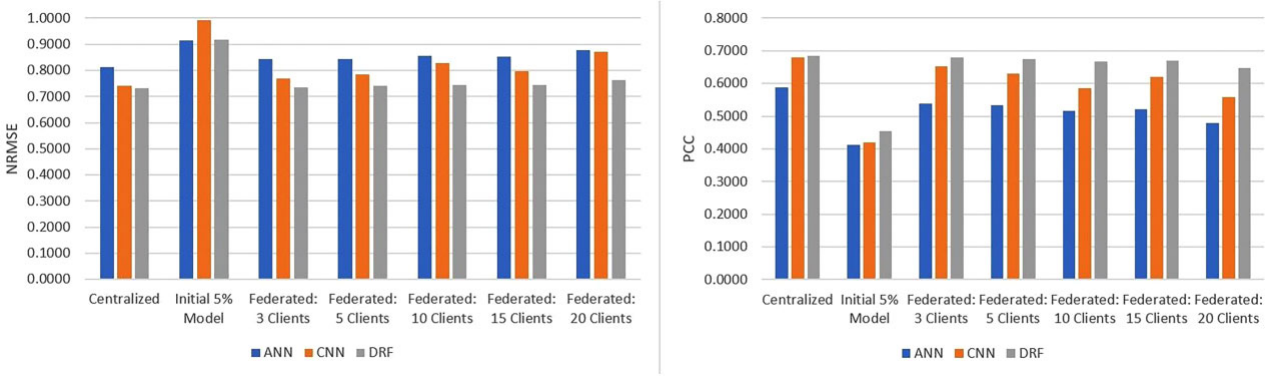
Average NCI-60 test results over three cell-lines with three different data partitions using ANN, REFINED-CNN and REFINED-DRF on NCI60 in terms of NRMSE and PCC

The National Cancer Institute (NCI) dataset consists of responses of over 55 000 chemicals screened on over 100 cancerous cell-lines. Details on the NCI dataset are included in the Supplementary Material. Out of 60 cell-lines available in NCI-60, we used 3 cell-lines that were exposed to most number of drugs. Each of these three selected cell-lines was evaluated with three different random realizations of $\left\{D_{T},D_{V},D_{H}\right\}$. Average predictive performance on these 3×3 cell-line-partition combination is reported in Figure 4 and Supplementary Table S4. Results on NCI-60 follow similar pattern as was observed on CCLE, with REFINED-DRF outperforming the ANN and REFINED-CNN in all of the centralized and federated settings, barring the initial 5% server model. The REFINED-DRF slightly outperformed the REFINED-CNN on the centralized model, but the performance improvement amplified as the number of clients increased. The REFINED-CNN and REFINED-DRF significantly outperformed the ANN, with REFINED-DRF achieving on average 0.089 lower NRMSE and 0.127 higher PCC. For all models, the performance degraded as the number of clients increased similar to the CCLE scenario.

Client-specific baseline models for NCI-60 were also run according to the procedure described for baseline CCLE models, with the exception that only A549 cell-line was tested since it had the highest sample size. We used the same model architectures and parameters as the federated models. We tested the performance of models with lower complexities, but such models did not contribute toward improvement in average predictive performance. The performance of the baseline model for cell-line A549 is shown in Supplementary Table S5. Clearly, the federated set up showed considerable improvement in predictive performance from the baseline individual client models. On an average, the NRMSE in federated setting were 6.3%, 11.6% and 15.8% lower than that of the baseline model for the ANN, REFINED-CNN and REFINED-DRF, respectively. The PCC under federated setting improved by 23.43%, 30.78% and 34.81% as compared to the baseline model, for the ANN, REFINED-CNN and REFINED-DRF, respectively. Again, we observed that REFINED-DRF offered highest improvement in federated setting as compared to its baseline counterpart.

### 3.3 Robustness analysis of federated setting

Customarily, robustness of federated setting is studied under various versions of adversarial attacks (Bagdasaryan *et al.*, 2020; Melis *et al.*, 2019; Nasr *et al.*, 2019). In this study, we analyze the robustness of our federated setting under non-malicious data swaps and quasi-random allocation of samples among clients. Studying robustness under data swaps was inspired by FDA’s Multi-omics Enabled Sample Mislabeling and Correction Challenge (Boja *et al.*, 2018) that solicited methodological developments to detect and correct mislabeled samples. We conjecture that in a federated setting, where clients are observing a homogeneous population, the totality of information collected by all clients would provide a comprehensive view of the population, which could help us accurately identify and resolve accidental data swaps and data mislabeling. In the demonstrative scenario below, we assume that the data swaps are completely random and no systematic effort was made by the clients to fabricate data.

In the second scenario, we investigate how well the federated setting—that we have designed here—can handle heterogeneity across the clients’ databases. A quasi-random allocation was designed to mimic situations where client’s database consists of population clusters and thus violates the assumption that each client is taking measurement on a homogeneously distributed population. This scenario also corresponds to splitting the data in a cognizant manner by taking into account the drug-target metadata.

### 3.4 Robustness under data swaps

**Fig. 5.**
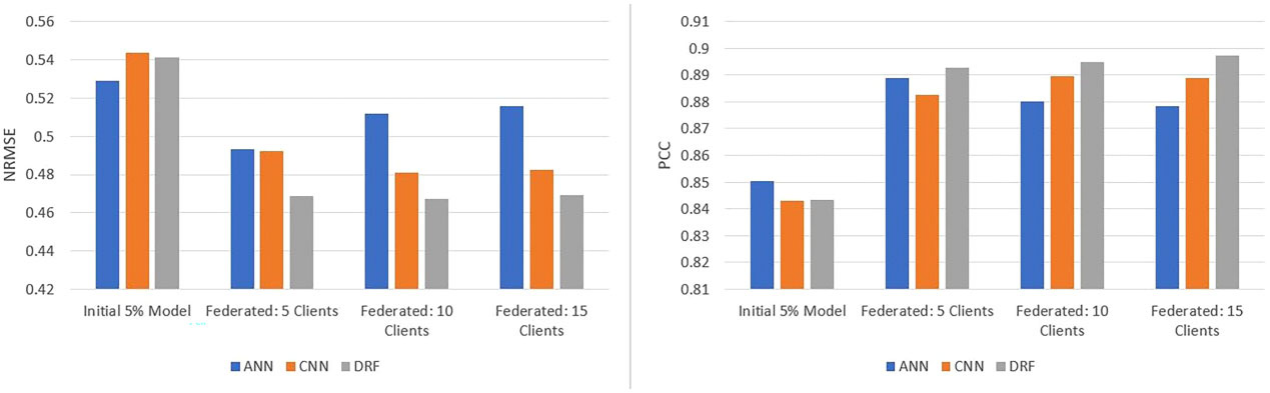
Average CCLE test results with data swaps over three different data partitions with ANN, REFINED-CNN and REFINED-DRF on CCLE in terms of NRMSE and PCC

To test the robustness against erroneous data entries, each client permuted 20% of their target values before training. In other words, the row indices of 20% of responses in $D_{T,j}^{\left(-I\right)}$ were randomly permuted for each j=1,2,..,n;n∈{5,10,15}. The experiment was run with the same parameters and model architectures as the federated experiments. Given a particular split of $D=\{D_{T},D_{V},D_{H}\}$, we deployed both the permuted and unpermuted versions of the federated model so that we can have a like-for-like comparison of the predictive performance. We present the average (over three splits) predictive performance of the permuted version of the federated learner on CCLE dataset in Figure 5. A comparison of Figure 5 with Figure 3 reveals, quite obviously, that the performance of all the competing models suffered under permutation. However, it was also apparent that the REFINED-DRF incurred least loss due to the permutation of target values. Robustness of conventional random forest against mislabeling of target classes was reported in Knights *et al.* (2011). It appears that DRF also inherits that robustness in federated setting as well.

### 3.5 Robustness under quasi-random allocation of samples

In this section, we consider the performance of the models in the federated setting where $D_{T}^{\left(-I\right)}$ was allocated to each client in a quasi-random fashion. Observe that, a CRD-based allocation of $D_{T,j}^{\left(-I\right)},j=1,2,..,n$ essentially implies that the observation units are homogeneously distributed and each client is equally likely to observe any observation unit. However, in several realistic situations, certain clients are more likely to observe certain clusters of observation units (e.g. conceptualize a hospital located in a disease hot spot and therefore only certain kinds of inhibitors dominate the set of drugs prescribed by that client). To mimic this real-world clustering behavior of observation units, we created scenarios where certain clients can observe certain drugs only, i.e. keeping the number of clients constant, we varied the number of unique drugs each client had access to. We tested the federated learner under several scenarios of this setting. On one end of the spectrum, we had a situation where clients’ drug sets were completely disjoint, while at the other end, we had all the clients containing observations on all the drugs. We deployed this scenario only on CCLE, because it had observations on 24 distinct drugs, in the following way:

First, we fixed the number of clients n=12 and introduced an overlapping parameter *k* that controlled the number of unique drugs per client. We allowed the following values of k∈{2,8,16,24}. When k=2, each client data corresponds to two unique drugs. Thus, k=2 was a no-overlap case, where clients’ drug sets were completely disjoint. In this situation, the drug–target metadata is a natural choice to split the data in a cognizant fashion. In our synthetic case, the drug set allocated to Client 0 (over three replicates) was dominated by the compound Nultin-3, which is a small molecule inhibitor of MDM2 (Arya *et al.*, 2010). On the other hand, the drug set allocated to Client 2 (over three replicates) was dominated by the compound PHA-665752, which is a c-MET inhibitor (Zhi *et al.*, 2018) and the drug set allocated to Client 10 (over three replicates) was dominated by the compound TKI-258, which is an FGFR, VEGFR inhibitor (Dubbelman *et al.*, 2012). For 2<k<24, each client was allocated a random sample of *k* drugs out of 24 available drugs. Since n=12, for k>2 clients’ drug sets were no longer disjoint. When more than one client had the same drug in their drug sets, the cell-line data was split evenly among the corresponding clients. Finally, when k=24, clients’ drug set completely overlapped with each other with each client having data from all the drugs.

**Fig. 6.**
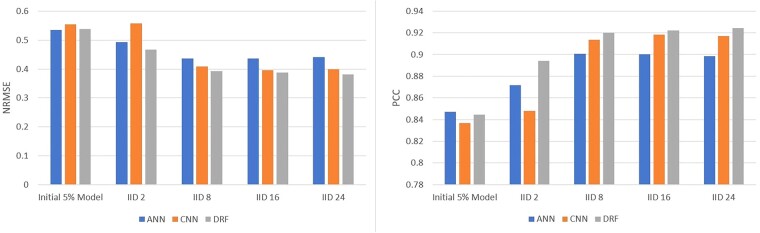
Average CCLE test results with quasi-random allocation of samples over three different data partitions with ANN, REFINED-CNN and REFINED-DRF on CCLE in terms of NRMSE and PCC

The predictive performance of the competing models is shown in Figure 6. Observe that the performance pattern of all the competing models remain fairly stable for k∈{8,16,24}. However, all the models performed poorly for k=2. As a matter of fact, REFINED-CNN struggled to converge in this extreme case even with a lower learning rate.

**Fig. 7.**
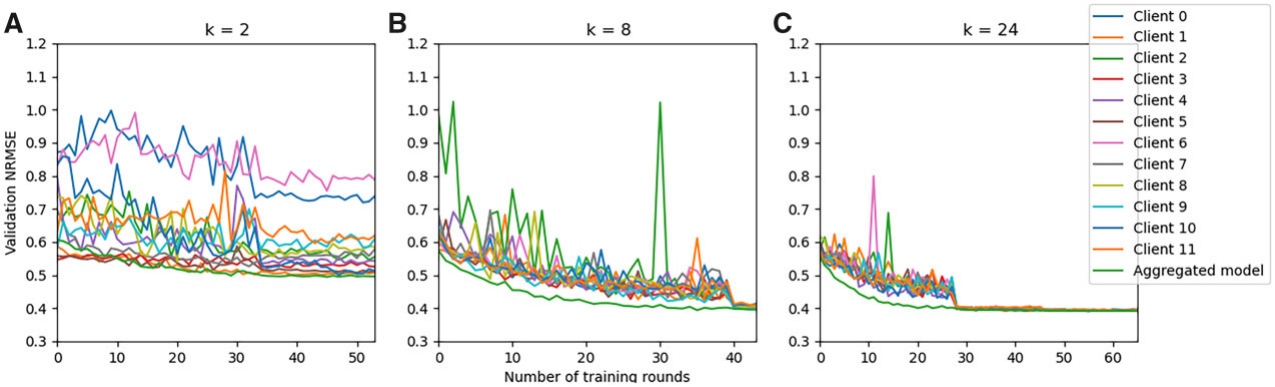
DRF validation NRMSE trajectories for (**A**) k=2, (**B**) k=8 and (**C**) k=24. The abscissa is allowed to vary for different *k* because each simulation stopped training at different training rounds based on the early stopping criteria

Given the observed sensitivity of the federated learner to clustered population (k=2), we proceed to further investigate whether it is possible to identify the existence of clusters from the information received from the clients. Recall, in our FL architecture, the server broadcasted the initial model to clients and received locally updated parameters from each client. The server also had access to $D_{V}$, which was randomly selected from the population. We conjecture that if $D_{T}^{\left(I\right)}$ and $D_{V}$ are both representative samples from the population, the server can track the validation loss to identify potential existence of clustered population among clients. To illustrate our point, we ask the server to calculate the validation loss for each client, on $D_{V}$, once it received locally updated parameters from them. Figure 7(A–C), offer the trajectory of validation loss for REFINED-DRF under k=2,8,24, respectively. Observe how the between-trajectory variance declined as *k* increased. Similar reduction in between-trajectory variances were observed for ANN and REFINED-CNN validation loss trajectories as well. Consequently, tracking the variation in validation loss across clients after each round of parameter update could potentially offer insight into the homogeneity of observational units across the clients. High variation in the validation loss trajectory would indicate presence of client-specific sub-populations.

We recommend a Gelman–Rubin (Gelman and Rubin, 1992) style convergence diagnostic measure to assess the mixing of client-specific validation loss trajectories. Suppose, there are *n* clients go over *M* rounds of parameter updates. Suppose, it was observed that m(≤M) updating rounds were required to achieve an approximate convergence in the validation loss trajectories among clients. Let $x_{1}^{\left(j\right)},x_{2}^{\left(j\right)},..x_{m-1}^{\left(j\right)}$ be observed trajectory of validation loss for client j,j=1,2,..n until the convergence point *m*. Define client-specific loss trajectory mean $\bar{x_{j}}=\frac{1}{m-1}\sum_{t=1}^{m-1}x_{t}^{\left(j\right)}$ and grand mean of validation loss as $\bar{x}=\frac{1}{n}\sum_{j=1}^{n}{\bar{x}}_{j}$. We can define between-trajectory variance $B=\frac{m-1}{n-1}\sum_{j=1}^{n}{\left({\bar{x}}_{j}-\bar{x}\right)}^{2}$ and within-trajectory variance $W_{j}=\frac{1}{m-2}\sum_{t=1}^{m-1}{\left(x_{t}^{\left(j\right)}-{\bar{x}}_{j}\right)}^{2}$. Define $W=\frac{1}{n}\sum_{j=1}^{n}W_{j}$. Then, a convex combination of *B* and *W*, with appropriate scaling, can be used to assess how well the client trajectories are mixing up to the convergence point *m*. For instance, once the within-trajectory and between-trajectory variances are defined, we can use $R=\frac{W}{\alpha B+(1-\alpha )W},\;\text{with}\;\alpha =1/(m-1)$ as a mixing diagnostic (Gelman and Rubin, 1992). Observe that, as B→0, then for reasonably large *m*, we have R≈1. This implies that for a given value of *m*, if *R* takes value in the neighborhood of 1, the client trajectories are mixing well and we can assume that the population is homogeneously distributed among clients. However, R≫1 (for a given *m*) would strongly indicate that clients are observing heterogeneous population clusters. Thus, the server can keep track of the *R* statistic to detect presence of clusters.

What should be done if clustering is detected by the server? The obvious option is to let each client train their own model and temporarily halt federated aggregation. In other words, the server only keeps track of the validation loss trajectories for each client without sending out aggregated parameter updates. The other option is to continue with federated updating but add a personalized layer in each client’s network. More specifically, each client uses the last layer of its network to *personalize* its own parameter estimates. Clients update their local parameters from the received aggregated model for all layers but the last layer, letting each client have independent parameters for personalization. Furthermore, each client records the number of non-improving epochs and the server averages them from all the clients so that the learning rate reduction and early stopping can still be performed. To compare the predictive capabilities of these two strategies, we conceptualize the following simulation study:

We examine the extreme case of no drug overlap among clients, i.e. k=2 on the CCLE dataset. We retain 10% of samples for each client for validation and another 10% are treated as hold-out sets. We allow each client to train its own model, without any federated updates, and test each client’s model on the foregoing test samples held out for each client. This predictive performance can be viewed as local predictive capability—the ability of each client model to predict observations drawn from the population cluster that the client is exposed to. We can also test the predictive performance of each client model on the entire test set obtained by concatenating the 10% samples held out for each client. This global predictive performance, observed on the global test set, is indicative of the efficacy of each client’s model on predicting test samples arising from population clusters that the client was not exposed to during training phase. Table 1 below shows the local and global predictive performance of individually trained REFINED-DRF and Federated REFINED-DRF with single personalized layer:

**Table 1.** Predictive performance of individually trained client REFINED-DRF and federated REFINED-DRF with a single personalization layer on both the local and global hold-out sets

Type of test set	Individual client DRF		Federated DRF with personalized layer	
	NRMSE	PCC	NRMSE	PCC
Local	0.74	0.64	0.72	0.69
Global	0.81	0.71	0.82	0.79

It is not reasonable to compare the local versus global predictive performances because of higher level of heterogeneity in the global test samples as compared to the local ones. Hence, we compare the local predictive performance of the two competing models separately from their global predictive performance. Clearly, the performance of individually trained models is comparable with the federated ones when the models are being tested on client-specific test data. However, when tested on global test set the PCC for Federated DRF is considerably higher as compared to that observed for individually trained DRF. This improvement is attributable to the federating process because the updates the clients are receiving from the server contain information about all the clients. Hence, there is an implicit information assimilation across clients.

## 4 Conclusion

In this article, we introduced a new FL framework that integrates DRFs with REFINED CNN with the goal of improving predictive performance in federated settings. Experimental results on two medical datasets using a federated simulation showed the proposed framework’s effectiveness in the cross-silo federated setting. The federated REFINED-DRF was shown to improve cross-validation error considerably as compared to local client models without any FL. We also found empirical evidence that REFINED-DRF outperformed the CNN and ANN on both datasets under federated and centralized settings. The conceptualized framework showed robustness against erroneous data entry and extreme population heterogeneity. We also found that the REFINED method was effective in the federated setting using only a subset of available data to train the REFINED mapping. Our methodology enjoyed a rich feature extraction protocol available due to the REFINED mapping and subsequent CNN-based convolutional operations. Then, it was paired with the ensemble of predictions available from DRFs. We also conceptualized a procedure for the server to detect presence of strong population heterogeneity across clients and developed a personalized formulation that could enable the clients to strike a balance between fully federated and fully individualized predictive frameworks. As such depending on the distance between the test case and the training cases, the clients can decide on whether to invoke personalized layer or use the conventional federated estimates. Clearly, the proposed federated REFINED-DRF framework is a viable approach for cross-silo horizontal FL from medical datasets. Future research will include development of a formal protocol that would enable clients to generate predictive distributions depending on the position of the test case vis-a-vis the position of the training cases in a REFINED map operating on samples and features simultaneously.

## Supplementary Material

vbad036_Supplementary_Data

## Data Availability

The CCLE gene expression and response data are available from the Dependency Map (DepMap) portal, at https://depmap.org/portal/download/. The NCI-60 identifier and response data can be downloaded from the National Cancer Institute, at https://wiki.nci.nih.gov/display/NCIDTPdata/NCI-60+Growth+Inhibition+Data.

## References

[vbad036-B1] Arya A.K. et al (2010) Nutlin-3, the small-molecule inhibitor of MDM2, promotes senescence and radiosensitises laryngeal carcinoma cells harbouring wild-type p53. Br. J. Cancer, 103, 186–195. 20588277 10.1038/sj.bjc.6605739 PMC2906734

[vbad036-B2] Bagdasaryan E. et al (2020) How to backdoor federated learning. In: Chiappa S., Calandra R. (eds) Proceedings of the Twenty Third International Conference on Artificial Intelligence and Statistics, Volume 108 of Proceedings of Machine Learning Research. pp. 2938–2948. PMLR.

[vbad036-B3] Bazgir O. et al (2020) Representation of features as images with neighborhood dependencies for compatibility with convolutional neural networks. Nat. Commun., 11, 1–13. 32873806 10.1038/s41467-020-18197-y PMC7463019

[vbad036-B4] Bazgir O. et al (2021) Investigation of REFINED CNN ensemble learning for anti-cancer drug sensitivity prediction. Bioinformatics, 37, i42–i50. 34252971 10.1093/bioinformatics/btab336 PMC8275339

[vbad036-B5] Boja E. et al (2018) Right data for right patient—a precisionFDDA NCI–CPTAC Multi-omics Mislabeling Challenge. Nat. Med., 24, 1301–1302. 30194412 10.1038/s41591-018-0180-x PMC6892367

[vbad036-B6] Chen H.-Y. et al (2022) On pre-training for federated learning*. arXiv preprint arXiv:2206.11488*.

[vbad036-B7] Dayan I. et al (2021) Federated learning for predicting clinical outcomes in patients with COVID-19. Nat. Med., 27, 1735–1743. 34526699 10.1038/s41591-021-01506-3 PMC9157510

[vbad036-B8] Ding Y. et al (2022) An efficient industrial federated learning framework for AIoT: a face recognition application. *arXiv preprint arXiv:2206.13398. *

[vbad036-B9] Dubbelman A.-C. et al (2012) Disposition and metabolism of 14C-dovitinib (TKI258), an inhibitor of FGFR and VEGFR, after oral administration in patients with advanced solid tumors. Cancer Chemother. Pharmacol., 70, 653–663. 23010851 10.1007/s00280-012-1947-2

[vbad036-B10] Feki I. et al (2021) Federated learning for CIVID-19 screening from Chest X-ray images. Appl. Soft Comput., 106, 107330. 33776607 10.1016/j.asoc.2021.107330 PMC7979273

[vbad036-B11] Gelman A. , Rubin D.B. (1992) Inference from iterative simulation using multiple sequences. Statist. Sci., 7, 457–472.

[vbad036-B12] Ghandi M. et al (2019) Next-generation characterization of the cancer cell line encyclopedia. Nature, 569, 503–508. 31068700 10.1038/s41586-019-1186-3 PMC6697103

[vbad036-B13] Katti K. et al (2022) MammoDL: mammographic breast density estimation using federated learning. *arXiv preprint arXiv:2206.05575.*

[vbad036-B14] Knights D. et al (2011) Supervised classification of microbiota mitigates mislabeling errors. ISME J., 5, 570–573. 20927137 10.1038/ismej.2010.148 PMC3105748

[vbad036-B15] Kontschieder P. et al (2015) Deep neural decision forests. In: *Proceedings of the IEEE International Conference on Computer Vision*, Santiago, Chile, pp. 1467–1475.

[vbad036-B16] Li D. , Wang J. (2019) FedMD: heterogenous federated learning via model distillation. *arXiv preprint arXiv:1910.03581.*

[vbad036-B28] Li T. et al (2020) Federated optimization in heterogeneous networks. In: *Proceedings of Machine learning and systems* 2, Austin, TX, USA, pp. 429–450.

[vbad036-B17] McMahan B. et al (2017) Communication-efficient learning of deep networks from decentralized data. In: *Artificial Intelligence and Statistics*, Ft. Lauderdale, FL, USA, pp. 1273–1282. PMLR.

[vbad036-B18] Melis L. et al (2019) Exploiting unintended feature leakage in collaborative learning. In: *2019 IEEE Symposium on Security and Privacy (SP)*, San Francisco, CA.

[vbad036-B19] Nasr M. et al (2019) Comprehensive privacy analysis of deep learning: passive and active white-box inference attacks against centralized and federated learning. In: *2019 IEEE Symposium on Security and Privacy (SP)*, San Francisco, CA, pp. 739–753. IEEE.

[vbad036-B20] Nguyen J. et al (2022) Where to begin? Exploring the impact of pre-training and initialization in federated learning. *arXiv preprint arXiv:2206.15387.*

[vbad036-B21] Pati S. et al (2021) The federated tumor segmentation (FeTS) challenge. *arXiv preprint arXiv:2105.05874.*

[vbad036-B22] Pati S. et al (2022a) Federated learning enables big data for rare cancer boundary detection. Nat. Commun., 13, 7346. 36470898 10.1038/s41467-022-33407-5 PMC9722782

[vbad036-B23] Pati S. et al (2022b) The federated tumor segmentation (FeTS) tool: an open-source solution to further solid tumor research. Phys. Med. Biol., 67, 204002. 10.1088/1361-6560/ac9449 PMC9592188 36137534

[vbad036-B24] Pfitzner B. et al (2021) Federated learning in a medical context: a systematic literature review. ACM Trans. Internet Technol., 21, 1–31.

[vbad036-B25] Reina A. et al (2021) OpenFL: an open-source framework for federated learning. *arXiv preprint arXiv:2105.06413.*

[vbad036-B26] Rieke N. et al (2020) The future of digital health with federated learning. NPJ Digit. Med., 3, 119. 33015372 10.1038/s41746-020-00323-1 PMC7490367

[vbad036-B27] Roth H.R. et al (2020) Federated learning for breast density classification: a real-world implementation. In: Domain Adaptation and Representation Transfer, and Distributed and Collaborative Learning: Second MICCAI Workshop, DART 2020, and First MICCAI Workshop, DCL 2020, Held in Conjunction with MICCAI 2020, Lima, Peru, October 4–8, 2020, Proceedings 2. Springer International Publishing, pp. 181–191.

[vbad036-B29] Sheller M.J. et al (2019) Multi-institutional deep learning modeling without sharing patient data: a feasibility study on brain tumor segmentation. In: Brainlesion: Glioma, Multiple Sclerosis, Stroke and Traumatic Brain Injuries: 4th International Workshop, BrainLes 2018, Held in Conjunction with MICCAI 2018, Granada, Spain, September 16, 2018, Revised Selected Papers, Part I 4. Springer International Publishing, pp. 92–104. 10.1007/978-3-030-11723-8_9 PMC6589345 31231720

[vbad036-B30] Sheller M.J. et al (2020) Federated learning in medicine: facilitating multi-institutional collaborations without sharing patient data. Sci. Rep., 10, 1–12. 32724046 10.1038/s41598-020-69250-1 PMC7387485

[vbad036-B31] Shen W. et al (2018) Deep regression forests for age estimation. In: *Proceedings of the IEEE Conference on Computer Vision and Pattern Recognition*, Salt Lake City, Utah, pp. 2304–2313.

[vbad036-B32] Shoemaker R.H. (2006) The NCI60 human tumour cell line anticancer drug screen. Nat. Rev. Cancer, 6, 813–823. 16990858 10.1038/nrc1951

[vbad036-B33] Sjöberg A. et al (2019) Federated learning of deep neural decision forests. In: International Conference on Machine Learning, Optimization, and Data Science, Tuscany, Italy, pp. 700–710. Springer.

[vbad036-B34] Tzinis E. et al (2021) Separate but together: unsupervised federated learning for speech enhancement from non-IID data. In: *2021 IEEE Workshop on Applications of Signal Processing to Audio and Acoustics (WASPAA)*, New Paltz, NY, USA, pp. 46–50. IEEE.

[vbad036-B35] Wang H. et al (2020) Federated learning with matched averaging. *arXiv preprint arXiv:2002.06440.*

[vbad036-B36] Xu J. et al (2021) Federated learning for healthcare informatics. J. Healthc. Inform. Res., 5, 1–19. 33204939 10.1007/s41666-020-00082-4 PMC7659898

[vbad036-B37] Yang T. et al (2018) Applied federated learning: improving google keyboard query suggestions. *arXiv preprint arXiv:1812.02903.*

[vbad036-B38] Zhao Y. et al (2018) Federated learning with non-IID data. *arXiv preprint arXiv:1806.00582.*

[vbad036-B39] Zhi J. et al (2018) Effects of PHA-665752 and vemurafenib combination treatment on in vitro and murine xenograft growth of human colorectal cancer cells with BRAFV600E mutations. Oncol. Lett., 15, 3904–3910. 29456739 10.3892/ol.2018.7770 PMC5795890

