# Supplementary material for: Federated learning framework integrating REFINED CNN and Deep Regression Forests
Source: Bioinform Adv. 2023 Mar 22;3(1):vbad036. doi: 10.1093/bioadv/vbad036 (PMC10074025; doi:10.1093/bioadv/vbad036)
Supplement: vbad036_Supplementary_Data [file vbad036_supplementary_data.pdf]

Supplementary Materials for Federated Learning Framework integrating REFINED CNN and Deep Regression Forests

Daniel Nolte[1], Omid Bazgir[1], Souparno Ghosh[2], Ranadip Pal[1]*

[1] *Dept. Electrical and Computer Engineering, Texas Tech University*
[2] *Department of Statistics, University of Nebraska - Lincoln*
ranadip.pal@ttu.edu

Data description and candidate models

Three types of federated learners were considered: (a) federated-ANN (refered to as ANN henceforth) with conventional feature vectors, (b) federated CNN using REFINED images (REFINED-CNN, henceforth), and (c) federated REFINED-DRF (REFINED-DRF, henceforth). Fully centralized counterparts of each of the competing models, trained on the complete dataset, were used for benchmarking the performance of the federated learners. Additionally, we considered two public datasets: (i) Cancer Cell Line Encyclopedia (CCLE), and (ii) NCI-60 to deploy the foregoing federated scenarios.

Datasets and Preprocessing

CCLE

The cancer cell line encyclopedia (CCLE) project is a collaborative effort between Broad institute, the Novartis Institutes for Biomedical Research, and the Genomics Institute of the Novartis Research Foundation to conduct a detailed genetic and pharmacologic characterization of a large panel of human cancer models [3, 5]. The dataset consists of responses elicited by 24 anticancer drugs screened on 504 different cancer cell lines with known genomic information. In our set up, we have two different inputs (a) PaDEL descriptors [8] of 24 anti-cancer drugs across 504 cell lines, and (b) microarray gene expression data for these 504 cell lines before drug application. The response consists of the area under the curve (AUC) of the drug responses for a particular drug applied on a particular cell line.

Each drug was originally characterized by 1444 chemical descriptors extracted from the PaDEL framework. After removing chemical descriptors that exhibit extreme zero-inflation (approximately 90% zeros) across the drugs, we ended up using 1072 features to characterize each drug in this dataset. Raw measurements of these 1072 drug descriptors were normalized between 0 and 1. Turning to omics data, expression profiles of 16,382 genes were used to characterize each cell line in CCLE dataset. As an initial screening we extracted only the features that exhibited non-trivial variability, across samples, as compared to the sum total of variabilities of all the

features. This unsupervised screening left us with 1101 genes to characterize each cell-line. The final dataset consisted of responses (AUC measurements) associated with 11,408 samples. The feature set corresponding to each sample consisted of 1101 gene expression and normalized values of 1072 chemical descriptors. Since the dataset has drug descriptors and gene expressions from two distinct, independent group of features, two separate REFINED mappings were generated and used. One mapping was performed on the drug descriptors and one on the cell line gene expressions resulting in two images for each observational unit. For the ANN case, the cell line and drug vectors were concatenated together to create the full feature vector for the drug-cell pair.

NCI-60

The US National Cancer Institute (NCI) began generating NCI-60, an anticancer drug screening on over 60 human tumor cell lines, in the 1980s for use as a drug discovery tool [7]. The dataset now consists of over 55,000 chemicals screened on over 100 cancerous cell lines. We used the unique NSC identifier associated with each compound in conjunction with the foregoing PaDEL software to extract the set of chemical descriptors for each compound. Afer removing compounds with missing descriptor values or more than 10% zero values, we ended up with 55,253 compounds screened on 159 cancerous cell lines. Each compound was characterized by a vector of 672 chemical descriptors. NCI-60 uses GI-50, the drug concentration resulting in 50% inhibition of cell proliferation, as the response of the cell line to an administered compound[7]. Since the dose administration protocol is logarithmic in nature, the negative log of GI-50 was used as the target for prediction, and the drug descriptors were used as the predictors. For illustration purpose, we arranged the cell-lines in descending order of the number of drugs they were exposed to and considered 3 cell-lines that occupied top 3 spots in the foregoing ranking scheme. We deploy the competing FL learners separately for each of these cell-lines. This reduced the computational burden and also ensured enough data availability for the training of deep models. The number of samples for each of the three evaluated cell lines are shown in Table 2. Observe that, our observational units in NCI-60 are characterized differently as compared to CCLE. Here, for each cell-line, the response is coupled with the chemical descriptor of the drug that produced the response. Whereas in CCLE we considered two classes of predictors (genetic information and drug chemical descriptors), for NCI-60 we only consider a single class of predictors (drug chemical descriptors)

Cell Line	A549/ATCC	OVCAR-8	SW-620
# of samples (drugs)	50857	50737	50722

Table S1. Number of samples for each of the three NCI-60 cell lines evaluated.

Simulation Setup

In this section we give details of how the federated learning framework was setup for two publicly available datasets that were considered herein. Subsequently, we offer the architectural details of the three aforementioned competing models.

Federated Learning Simulation

Since we worked on publicly available data, a synthetic federated learning protocol was conceptualized in following way:

- A total of $n + 1$ synthetic agents were generated in single device. A randomly chosen agent was designated to be the server, the remaining n agents were designated to be the clients. The server agent was assigned to initialize the predictive model, disseminate the initial model to the clients, and perform federated averages of parameters obtained from the clients. The client agents used the initial model shared by the server, fitted the model to their local data, updated the parameters conditional on their local dataset and returned locally updated parameters to the server. We created multiple federation sizes by varying n from 3 to 20.

- Data were assigned to each agent using random partition of focal dataset. Let $\mathcal{D} = \{\mathcal{D}_i\}_{i=1}^{N}$ be the N samples obtained from the foregoing datasets ($N = 11,408$ for CCLE and N ranged from 50,722-50,857 for NCI-60), with $\mathcal{D}_i = (Y_i, \boldsymbol{X}_i)$, where Y denotes the drug response and $\boldsymbol{X}$ denote the values of the predictor set. First, $\mathcal{D}$ was randomly partitioned into $\mathcal{D}_T \cup \mathcal{D}_V \cup \mathcal{D}_H$. The training set, $\mathcal{D}_T$, consisted of 80% of the data. The validation set $\mathcal{D}_V$ had 10% of the data and a holdout test dataset, $\mathcal{D}_H$, was allocated the remaining 10% of the data.

- Only the server had access to $\mathcal{D}_V$. $\mathcal{D}_H$ was held outside the FL loop to test the performance of the federated learner. $\mathcal{D}_T$ was randomly allocated to $n + 1$ agents in the following way. A randomly chosen 5% of $\mathcal{D}_T$ was allocated to the server to initialize the federated learner. We denote this set by $\mathcal{D}_T^{(I)}$. The remaining 95% of the data in $\mathcal{D}_T$, denoted by $\mathcal{D}_T^{(-I)}$, was randomly allocated to n clients using an approximately balanced completely randomized design (CRD). We experimented with more designed allocation in the robustness analysis section where drug-target information turned out to be natural metadata to determine data split in a more cognizant fashion.

- To alleviate effect of a single CRD, we performed 3 independent CRD allocations and predictions were averaged across these 3 allocations. When performing robustness analysis with designed drug allocation, we strived to enforce that certain drugs dominate the set of drugs allocated to the clients. For example, under the non-overlapping scenario, the drug set allocated to the Client 0 (over three replicates) was dominated by the drug Nultin-3 which is a small molecule inhibitor of MDM2 [1]. On the other hand, the drug set allocated to Client 2 (over three replicates) was dominated by the compound PHA-665752 which is a c-MET inhibitor [9], and the drug set allocated to Client 10 (over three replicates) was dominated by the compound TKI-258 which is an FGFR, VEGFR inhibitor [4].

While dealing with REFINED-DRF or REFINED-CNN, the server generated the REFINED mappings using $\mathcal{D}_T^{(I)}$ and transmitted this map to all the clients so that each client uses the same mapping, and the models are trained with consistent feature maps. Observe that, the server actually transmitted 2D spatial coordinates associated with each feature to the clients and therefore there was no leakage of observed values of these features. To add another layer of privacy, geomasking techniques could also be applied [6] on top of REFINED maps. However, we do not pursue deployment of masking in this article.

For all candidate models (ANN, REFINED-DRF, REFINED-CNN), the federated learning process started with the server training an initial model on $\mathcal{D}_T^{(I)}$. The initial model was then sent

to each of the n clients to initialize their local models on their share of $\mathcal{D}_T^{(-I)}$ ($\mathcal{D}_{T,j}^{(-I)}, j = 1, 2, ..., n$, say). Each client trained its model for E epochs on $\mathcal{D}_{T,j}^{(-I)}$ and sent the locally updated model parameters along with their respective number of training samples back to the server. Once all the clients had returned their model parameters, Federated Averaging was performed by the server and the averaged parameters were broadcasted to all the clients. This exchange of model parameters was repeated by the server and clients until a stopping criterion was reached.

We used the following early stopping rule: the server evaluated the model corresponding to the federally averaged parameters after each sweep of the training round on $\mathcal{D}_V$. If no improvement in validation loss was observed for 5 consecutive epochs, the learning rate was multiplied by a factor of 0.1 and broadcasted to all clients. The clients continued training with the new learning rate and resend locally updated parameters back to the server. The server performed the federated averaging and computed the validation loss on $\mathcal{D}_V$. If there was no improvement in validation loss for 10 consecutive epochs, the training process was halted. Adam optimizer was used in each model with a batch size of 100. Once the models finished training, they were evaluated on $\mathcal{D}_H$.

In the benchmark centralized version, only one agent was used. The agent trained the model on $\mathcal{D}_T$ (no further partition of $\mathcal{D}_T^{(I)}$ and $\mathcal{D}_T^{(-I)}$ was required), used $\mathcal{D}_V$ to compute validation loss and then used $\mathcal{D}_H$ to test the model performance. Obviously, the centralized and federated models were trained and evaluated on the same training/validation/test datasets for fair comparison.

The entire above-decribed process was replicated with three different random data splits of $\mathcal{D}$ into $\{\mathcal{D}_T, \mathcal{D}_V, \mathcal{D}_H\}$ and the results were averaged to evaluate the model performance.

A general overview of the process is shown in Algorithm 1.

Model architecture

The architecture of each model differed slightly across the datasets. The ANN and CNN architectures were optimized using a grid search for each dataset in our original REFINED-CNN paper [2]. The DRF architecture was based on the optimized CNN architecture with minor adjustments for each dataset. We also conducted a basic ablation test with minor changes in the number of convolutional layers, linear layers, and nodes and the results indicate robustness of the REFINED-DRF architecture. Below we offer detailed architectural parameters for ANN, REFINED-CNN and REFINED-DRF for each dataset.

ANN

The ANN architecture for CLLE used 7 fully connected layers with ReLU activation functions. The seven fully connected layers had 1500, 1000, 600, 300, 100, 50, and 1 neuron/s, respectively. A dropout layer was added before the last layer with a drop probability of 50%. The centralized and federated learning rates were set to 1×10^{-3}.

For NCI-60 ANN, only 6 fully connected linear layers with ReLU activation's were used. No dropout layers were included for the NCI-60 ANN. The six fully connected layers had neuron sizes of 1000, 800, 500, 200, 100, and 1, respectively. The learning rate for the NCI-60 ANN was set to 2.5×10^{-4} for both the federated and centralized cases.

Algorithm 1 Federated DRFs with REFINED mapping

Require: server, S, and n clients each with their local datasets, $\mathrm{d_i}$, where $\mathrm{d_0}$ is the server set

```
Server Executes:
  Initialize CNN & tree parameters for GlobalModel
  Learn REFINED mapping, R, from d_0
  Map d_0 into images using R
  for each client i ∈ 1, 2, ..., n do
    Send R to client
    Client maps local dataset, d_i, into images using R
  end for
  while not converged do
    GlobalModel = trainDRF(GlobalModel, d_0)
  end while
  while not converged do
    Updates = {}
    for each client i ∈ 1, 2, ..., n do
      Send GlobalModel to client i
      Client updates their LocalModel with received GlobalModel
      Client executes ClientUpdate := trainDRF(LocalModel, d_i)
      Client returns ClientUpdate
      Updates = Updates ∪ ClientUpdate
    end for
    GlobalModel = ServerAggregation(Updates)
  end while
trainDRF: GlobalModel, d_i
  for batch jj ∈ d_i split into batches do
    Update CNN parameters with leaf node parameters stationary
  end for
  Update all leaf nodes mean and covariance with CNN parameters stationary
```

REFINED-CNN

Since the CCLE dataset contains two images per sample, a hybrid CNN architecture was adopted where one arm of the CNN processed the first image, the second arm processed the second image, and the two output feature maps were flattened and concatenated together. The detailed model architecture is shown in Supplementary Fig. S1. The learning rate used for the centralized and federated cases was 1×10^{-4} with the Adam optimizer. A lower learning rate had to be adopted for the CNN compared to the ANN and DRF because the model wasn't consistently converging in the federating setting with over 10 clients.

Unlike CCLE, the NCI-60 dataset consisted of just drug descriptor features, so just a single REFINED mapping was required and each sample contained a single image. Supplementary Fig. S2 shows the detailed model architecture used for this scenario. A learning rate of 2.5×10^{-4} was used along with the Adam optimizer for both the centralized and federated cases.

REFINED-DRF

The DRFs were constructed using a CNN similar to the one used for the CNN model, with the CNN outputs connected to the trees in the forest. The CCLE DRFs CNN architecture was similar to the regular CNN architecture, except that an additional linear layer was included, and the output of the CNN was 150 features to use for the routing of the trees rather than the single regression value it typically is. A learning rate of 1×10^{-3} was used for both the centralized and federated cases.

The NCI-60 DRF CNN architecture was also changed slightly for more improved results. The output size of 256 was chosen for the routing probabilities of the trees and the convolutional filter size was changed from 7×7 to 5×5. The fully connected layers had increased neuron sizes as well. A learning rate of 2.5×10^{-4} was used for the centralized and federated cases.

The ANN and CNN architectures were optimized using a grid search for each dataset. The DRF architecture was based on the CNN optimized architecture with minor adjustments for each dataset. The detailed CCLE and NCI-60 DRF model architectures can be seen in the Supplementary Figures S1 and S2. Compared to the CNN architectures, an additional fully connected layer and different neuron sizes were selected for the DRF architecture based on the improved performance with the additional layer. The fully connected neuron sizes were altered to better match the output size of the forest. The forest itself consisted of 10 trees, each with a depth of 7. At the end of each epoch, with the network parameters stationary, the leaf node updates were iterated 10 times using all of the data included in the epoch.

SUPPLEMENTARY TABLES

Model Type	ANN		CNN		DRF	
	NRMSE	PCC	NRMSE	PCC	NRMSE	PCC
Centralized	0.3821	0.9243	0.3675	0.9301	**0.3665**	**0.9306**
Initial 5% Model	**0.5386**	**0.8519**	0.5457	0.8430	0.5559	0.8334
Federated: 3 Clients	0.4021	0.9161	0.3885	0.9219	**0.3708**	**0.9291**
Federated: 5 Clients	0.4232	0.9073	0.3951	0.9192	**0.3844**	**0.9236**
Federated: 10 Clients	0.4215	0.9082	0.4074	0.9138	**0.3828**	**0.9245**
Federated: 15 Clients	0.4399	0.8997	0.4065	0.9147	**0.3890**	**0.9221**
Federated: 20 Clients	0.4523	0.8936	0.4144	0.9113	**0.3920**	**0.9207**

Table S2. Average test results over three different data partitions with ANN, REFINED-CNN, and REFINED-DRF on CCLE in terms of NRMSE and PCC.

Model Type	ANN		CNN		DRF	
	NRMSE	PCC	NRMSE	PCC	NRMSE	PCC
3 Clients	0.4459	0.8956	0.4498	0.8940	0.4640	0.8865
5 Clients	0.4754	0.8816	0.4783	0.8795	0.4853	0.8751
10 Clients	0.5029	0.8680	0.5177	0.8588	0.5163	0.8578
15 Clients	0.5207	0.8586	0.5421	0.8458	0.5338	0.8475
20 Clients	0.5348	0.8513	0.5574	0.8381	0.5465	0.8396
Average Federated Increase	0.0682	0.0339	0.1067	0.0530	0.1254	0.0627
Average % Federated Increase	13.59%	3.92%	20.60%	6.19%	24.42%	7.32%

Table S3. Average test results for individual clients among three different data partitions with ANN, REFINED-CNN, and REFINED-DRF on CCLE in terms of NRMSE and PCC.

Model Type	ANN		CNN		DRF	
	NRMSE	PCC	NRMSE	PCC	NRMSE	PCC
Centralized	0.8118	0.5879	0.7414	0.6788	**0.7333**	**0.6853**
Initial 5% Model	**0.9134**	0.4112	0.9937	0.4186	0.9175	**0.4537**
Federated: 3 Clients	0.8423	0.5386	0.7693	0.6518	**0.7344**	**0.6803**
Federated: 5 Clients	0.8450	0.5347	0.7844	0.6302	**0.7399**	**0.6739**
Federated: 10 Clients	0.8555	0.5172	0.8267	0.5867	**0.7446**	**0.6687**
Federated: 15 Clients	0.8524	0.5222	0.7984	0.6214	**0.7444**	**0.6688**
Federated: 20 Clients	0.8779	0.4782	0.8706	0.5576	**0.7641**	**0.6476**

Table S4. Average test results over three cell lines with three different data partitions using ANN, REFINED-CNN, and REFINED-DRF on NCI60 in terms of NRMSE and PCC.

Model Type	ANN		CNN		DRF	
	NRMSE	PCC	NRMSE	PCC	NRMSE	PCC
3 Clients	0.8623	0.5080	0.8293	0.5703	0.8180	0.5868
5 Clients	0.8784	0.4809	0.8680	0.5245	0.8504	0.5455
10 Clients	0.9058	0.4280	0.9158	0.4645	0.8836	0.4958
15 Clients	0.9161	0.4080	0.9364	0.4387	0.9034	0.4672
20 Clients	0.9250	0.3850	0.9570	0.4128	0.9167	0.4446
Average Federated Increase	0.0569	0.0992	0.1055	0.1427	0.1388	0.1707
Average % Federated Increase	6.29%	23.43%	11.61%	30.78%	15.77%	34.81%

Table S5. Average test results for individual clients among three different data partitions with ANN, REFINED-CNN, and REFINED-DRF on CCLE in terms of NRMSE and PCC.

SUPPLEMENTARY FIGURES

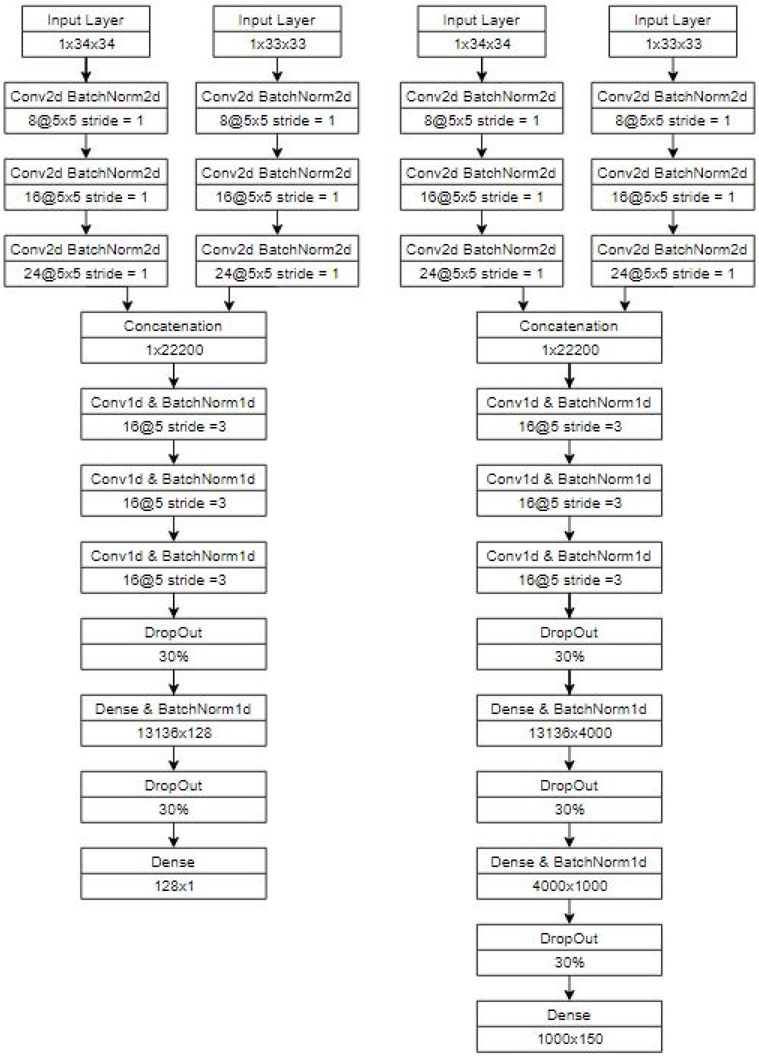

Fig. 1. CCLE CNN (left) and DRF (right) model architectures, each layer is followed by a ReLU activation function besides the input, concatenation, and dropout layers.

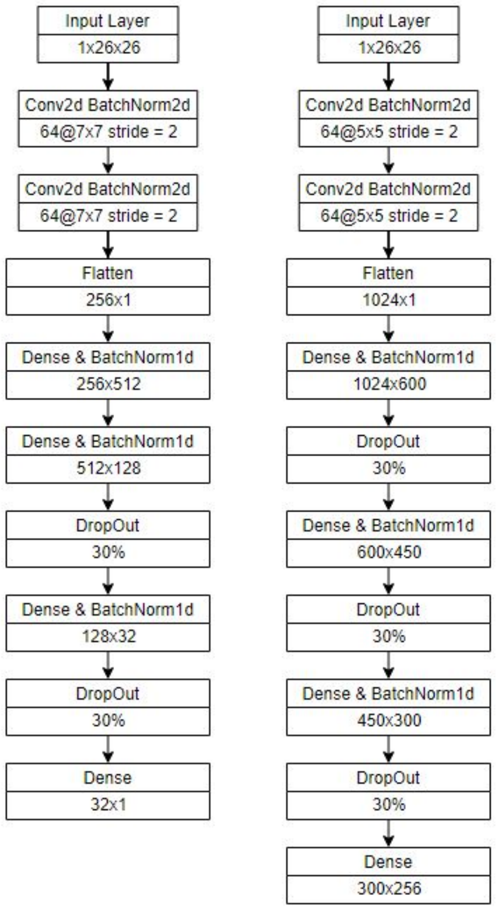

Fig. 2. NCI60 CNN (left) and DRF (right) model architectures, each layer is followed by a ReLU activation function besides the input, flatten, and dropout layers.

References

A K Arya et al. Nutlin-3, the small-molecule inhibitor of mdm2, promotes senescence and radiosensitises laryngeal carcinoma cells harbouring wild-type p53. *British Journal of Cancer*, 103:186–195, 2010.

Omid Bazgir et al. Representation of features as images with neighborhood dependencies for compatibility with convolutional neural networks. *Nature communications*, 11(1):1–13, 2020.

Cancer Cell Line Encyclopedia Consortium and Genomics of Drug Sensitivity in Cancer Consortium. Pharmacogenomic agreement between two cancer cell line data sets. *Nature*, 528(7580):84, 2015.

Anne-Charlotte Dubbelman et al. Disposition and metabolism of 14c-dovitinib (tki258), an inhibitor of fgfr and vegfr, after oral administration in patients with advanced solid tumors. *Cancer chemotherapy and pharmacology*, 70, 2012.

Mahmoud Ghandi et al. Next-generation characterization of the cancer cell line encyclopedia. *Nature*, 569(7757):503–508, 2019.

Jill E Sherman and Tamara L Fetters. Confidentiality concerns with mapping survey data in reproductive health research. *Studies in family planning*, 38(4):309–321, 2007.

Robert H Shoemaker. The nci60 human tumour cell line anticancer drug screen. *Nature Reviews Cancer*, 6(10):813–823, 2006.

Chun Wei Yap. Padel-descriptor: An open source software to calculate molecular descriptors and fingerprints. *Journal of computational chemistry*, 32(7):1466–1474, 2011.

Jie Zhi et al. Effects of pha-665752 and vemurafenib combination treatment on in vitro and murine xenograft growth of human colorectal cancer cells with brafv600e mutations. *Oncology letters*, 15, 2018.
